# Transcriptional regulation of chicken leukocyte cell-derived chemotaxin 2 in response to toll-like receptor 3 stimulation

**DOI:** 10.5713/ajas.19.0192

**Published:** 2019-07-02

**Authors:** Seokhyun Lee, Ra Ham Lee, Sung-Jo Kim, Hak-Kyo Lee, Chong-Sam Na, Ki-Duk Song

**Affiliations:** 1 Department of Animal Biotechnology, College of Agricultural and Life Sciences, Jeonbuk National University, Jeonju 54896, Korea; 2 Department of Biotechnology, Hoseo University, Asan 31499, Korea; 3 The Animal Molecular Genetics and Breeding Center, Jeonbuk National University, Jeonju 54896, Korea

**Keywords:** Chicken, Leukocyte Cell-derived Chemotaxin 2 (LECT2), Gene Expression, Innate Immune Receptor Signaling

## Abstract

**Objective:**

Leukocyte cell-derived chemotaxin 2 (LECT2) is associated with several physiological processes including inflammation, tumorigenesis, and natural killer T cell generation. Chicken *LECT2* (*chLECT2*) gene was originally identified as one of the differentially expressed genes in chicken kidney tissue, where the chickens were fed with different calcium doses. In this study, the molecular characteristics and gene expression of *chLECT2* were analyzed under the stimulation of toll-like receptor 3 (TLR3) ligand to understand the involvement of *chLECT2* expression in chicken metabolic disorders.

**Methods:**

Amino acid sequence of LECT2 proteins from various species including fowl, fish, and mammal were retrieved from the Ensembl database and subjected to Insilco analyses. In addition, the time- and dose-dependent expression of *chLECT2* was examined in DF-1 cells which were stimulated with polyinosinic:polycytidylic acid (poly [I:C]), a TLR3 ligand. Further, to explore the transcription factors required for the transcription of *chLECT2*, DF-1 cells were treated with poly (I:C) in the presence or absence of the nuclear factor κB (NFκB) and activated protein 1 (AP-1) inhibitors.

**Results:**

The amino acid sequence prediction of chLECT2 protein revealed that along with duck LECT2 (duLECT2), it has unique signal peptide different from other vertebrate orthologs, and only chLECT2 and duLECT2 have an additional 157 and 161 amino acids on their carboxyl terminus, respectively. Phylogenetic analysis suggested that chLECT2 is evolved from a common ancestor along with the actinopterygii hence, more closely related than to the mammals. Our quantitative polymerase chain reaction results showed that, the expression of *chLECT2* was up-regulated significantly in DF-1 cells under the stimulation of poly (I:C) (p<0.05). However, in the presence of NFκB or AP-1 inhibitors, the expression of *chLECT2* is suppressed suggesting that both NFκB and AP-1 transcription factors are required for the induction of *chLECT2* expression.

**Conclusion:**

The present results suggest that *chLECT2* gene might be a target gene of TLR3 signaling. For the future, the expression pattern or molecular mechanism of chLECT2 under stimulation of other innate immune receptors shall be studied. The protein function of chLECT2 will be more clearly understood if further investigation about the mechanism of LECT2 in TLR pathways is conducted.

## INTRODUCTION

Leukocyte cell-derived chemotaxin 2 (LECT2) is a multifunctional protein secreted from liver, and a chemotactic factor for neutrophils. Human LECT2 has a molecular mass of ~16 kDa [1], with three intramolecular disulfide bonds [2]. The amino acid sequence has been well conserved throughout evolution. Accumulating evidence indicates that, LECT2 is associated with several physiologic functions including regulation of liver regeneration [3], tumorigenesis [4,5], suppression of inflammatory arthritis and cytokine production [6,7] and hepatic natural killer T (NKT) cells generation [8]. It is reported that plasma LECT2 concentration of patients with bacterial sepsis is significantly lower than healthy humans and LECT2 expression is correlated with systemic inflammation [9]. In *LECT2*-knockout mice, hepatitis gets more severe due to the increased number of NKT cells and their cytokine ligands, and therefore, the homeostasis of NKT cells in the liver is negatively regulated by LECT2 [8,10]. It was also reported that LECT2 has anti-inflammatory and tumor-suppressive actions in oncogenic β-catenin–induced liver tumorigenesis [5]. Human LECT2 is associated with inflammation and insulin resistance and the suppression of inflammatory and immune responses by LECT2 can inhibit tumorigenesis in liver [11,12].

In chicken, LECT2 was identified as *mim-1* protein, P33, which is chemotactic for heterophils [13], and it also activates antimicrobial responses toward *Salmonella enteritidis* from chicken heterophils [13]. In *Salmonella enteritidis* infected chickens, the level of LECT2 protein was decreased in heterophils compared to macrophages without difference at RNA level [14,15]. In addition, the expression of duck *LECT2* was significantly increased in liver and spleen by duck hepatitis virus type I infection [16], or the treatment of polyinosinic-polycytidylic acid (poly [I:C]), implying the LECT2 may play a role in the defense mechanism against viral infections in duck and be regulated by innate immune receptor signaling pathways, i.e., toll-like receptors 3 (TLR3).

The recognition of the microbial molecular patterns by TLRs trigger the intracellular signaling cascades where transcription factors, such as nuclear factor κB (NFκB) and activated protein 1 (AP-1) are activated for the transcriptional regulation of target genes [17]. In chicken, flagellin and lipopolysaccharide, which are bacterial products, activate both NFκB and AP-1 signaling pathways; the engagements of TLRs-TLR ligands lead to the degradation of the inhibitor of NFκB (IκB) to allow the release of NFκB for translocation into the nucleus, and activate mitogen-activated protein kinase to phosphorylate AP-1, leading to the binding to the promoter of the target genes by activated NFκB and AP-1 [18,19].

In this study, we evaluated transcriptional regulation of chicken LECT2 (chLECT2) in response to a TLR3 ligand engagements and explored signaling pathways responsible for the transcription of *chLEC2* with the inhibitors of NFκB and AP-1 in chicken DF-1 cells.

## MATERIALS AND METHODS

### Bioinformatics analysis

The LECT2 amino acid sequences of various species (chicken, duck, zebrafish, asian seabass, mouse, rat, pig, cow, human, and chimpanzee) were retrieved from the Ensembl database (http://www.ensembl.org/) (Table 1). The amino acid sequences were aligned with the ClustalW implemented in the BioEdit tool. The protein domains were predicted by using the SMART domain search program (http://smart.embl-heidelberg.de/). Phylogenetic analyses were performed with MEGA7 software [20].

### Cell culture

The chicken DF-1 cell line was purchased from the American Tissue Culture Collection (CRL-12203, Manassas, VA, USA). DF-1 cells were cultured in Dulbecco’s modified eagle medium, supplemented with 10% fetal bovine serum, 2 mM L-glutamine and 100 U/mL each of penicillin and streptomycin (Thermo Scientific, Logan, UT, USA) at 37°C in a humidified atmosphere of 5% CO_2_.

### Stimulation of DF-1 cells by TLR3 ligand and inhibitors of NFκB and AP-1

Poly (I:C) (TLR3 ligand) was purchased from InVivogen (San Diego, CA, USA) and dissolved in endotoxin and nuclease free water and kept at −20°C until use. DF-1 cells were treated at the concentrations of 0.1, 1, 5, and 10 μg/mL for 24 h. Also, to determine the time kinetics of mRNA expression, cells were treated for 1, 3, 6, 12, and 24 h at 10 μg/mL concentration. BAY 11–7085 and Tanshione IIA were purchased from Sigma-Aldrich (Louis, MO, USA). BAY 11-7085 (BAY) inhibits the IκBα phosphorylation, resulting in prevention of NFκB activation, was used as NFκB inhibitor. Tanshinone IIA (Tan-II) may inhibit the binding of AP-1 to DNA in chicken heteophils [19]. DF-1 cells were pretreated with BAY 11–7085 (5 μM) and Tan-II (25 μM), for 3 h, of which concentrations and time were determined from the preliminary experiment in this study, and then stimulated with poly (I:C) (5 μg/mL) for 3 h.

### RNA extraction and cDNA preparation

Trizol (Invitrogen, Carlsbad, CA, USA) was used to extract total RNA from chicken DF-1 cells. Total RNA was quantified by NanoDrop spectrophotometer (Thermo Fisher Scientific Inc., Waltham, MA, USA). After quantification, 2 μg of total RNA was reverse-transcribed to synthesize cDNA using the using QuantiTect Reverse Transcription Kit (Toyobo, Osaka, Japan) according to the manufacturer’s instructions.

### Quantitative real-time polymerase chain reaction analysis

Quantitative real-time polymerase chain reaction (qRT-PCR) was conducted with a CFX-96 RT-PCR detection system (BioRad, Hercules, CA, USA) for the analysis of *chLECT2* expression. The qRT-PCR conditions were as follows: an initial step at 94°C for 3 min; 39 cycles at 94°C for 10 s, 58°C for 30 s, and 72°C for 30 s; and a final step at 72°C for 10 min. Dissociation was performed at 0.5°C increments from 55°C to 95°C for over 25 min. The primer sequences of the chicken interleukin 1B (*chIL1B*), *chLECT2* and the glyceraldehyde 3-phosphate dehydrogenase (GAPDH) used in this study are shown in Table 2. All samples were measured in triplicates to ensure reproducibility, and Ct values were calculated by the 2^−ΔΔCt^ method [21], to calculate the relative expression level of mRNA on each sample, which was expressed as a ratio relative to *GAPDH* expression.

### Statistical analysis

Results were presented as means±standard deviation of triplicate independent experiments. Statistical significance was assessed using a Student’s t-test. A value p<0.05, as compared with the non-treated control, was considered statistically significant.

## RESULTS

### Phylogenetic analysis of leukocyte cell-derived chemotaxin 2 sequences

**Figure 1:**
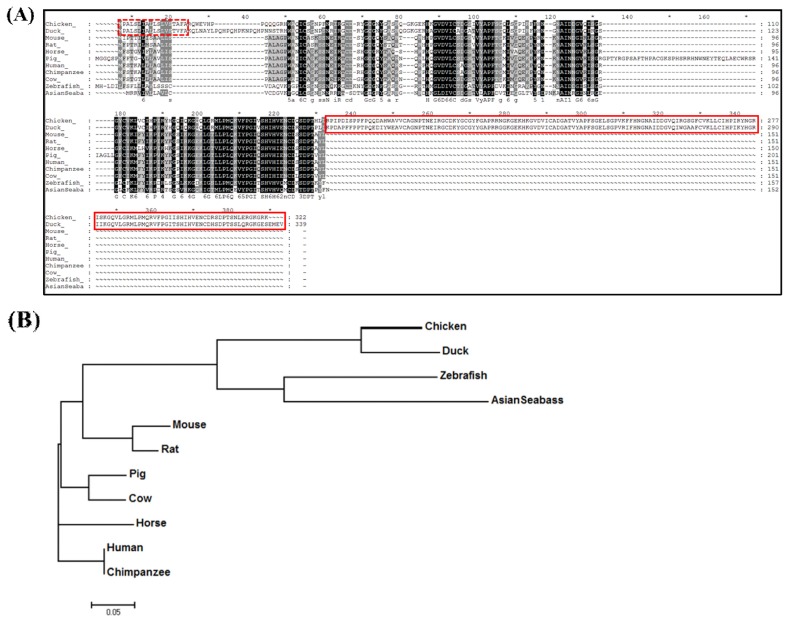
Analysis of LECT2 amino acid sequences. (A) Comparison of amino acid sequences of chicken LECT2 in various species (chicken, duck, zebrafish, Asian seabass, mouse, rat, pig, cow, horse, human, chimpanzee). Only chickens and ducks have the same signal peptide (dotted box) starting at position 1 and ending at position 18. In addition, the chicken and duck have unique 157 and 161 amino acids compared to other species (solid box). (B) Phylogenetic tree of LECT2 in various species. Phylogenetic analyses were performed with the amino acid sequence of each species by MEGA7 software method. ChLECT2 was clustered in the same clade with duck and fish group. The bar indicates 5% amino acid divergence. LECT2, leukocyte cell-derived chemotaxin 2; chLECT2, chicken LECT2.

The amino acid sequence of chLECT2 was analyzed and compared with other species. The *chLECT2* gene sequence was identified as a differentially expressed gene from chicken kidney RNA-seq study, where chickens were fed with different doses of calcium [22]. To verify and compare the chLECT2 amino acid sequences with other vertebrates, the amino acid sequences of LECT2 orthologs from fowl, fishes and mammal including duck, zebrafish, Asian seabass, mouse, rat, pig, cow, human and chimpanzee) were retrieved from ensemble database and aligned (Figure 1A). The analysis with SignalP v4.0 program (http://www.cbs.dtu.dk/) showed that chLECT2 and duck LECT2 (duLECT2) had the same signal peptide whose amino acid sequences starts at position 1 and ends at position 18. Besides, chLECT2 and duLECT2 had additional amino acids on carboxy terminus (157 for chicken and 161 amino acids for duck) compared to other species. The evolutionary relationships between chLECT2 and LECT2 orthologs were analyzed using phylogenetic tree (Figure 1). Phylogenetic analysis suggested that chLECT2 is evolved from a common ancestor with ducks. Also, it exhibited that LECT2 from fowls is relatively closely related that from fishes, while far from mammalian LECT2s.

### *chLECT2* expression from DF-1 cells in response to TLR3 ligand stimulation

**Figure 2:**
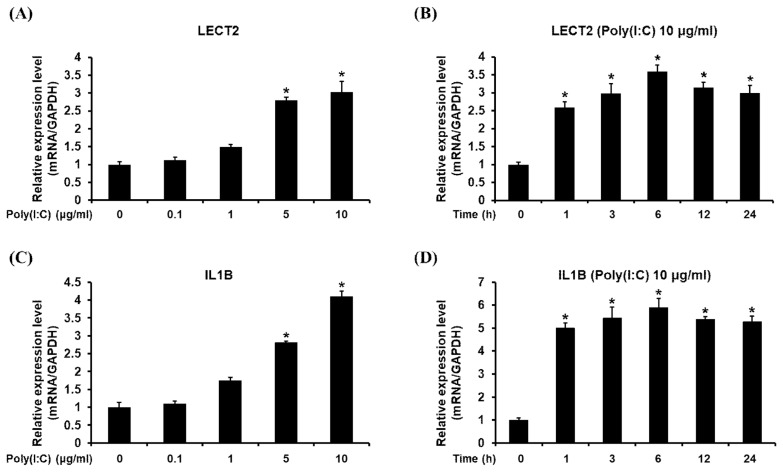
Analysis of expression levels of *chLECT2* and *chIL1B* in the cultured chicken DF-1 fibroblast cells with the treatment of different concentration and time of poly (I:C). (A) and (C) Chicken DF-1 cells were treated with 0.1, 1, 5, and 10 μg/mL concentrations of poly (I:C) for 24 h, and the expressions of *chLECT2* and *chIL1B* was analyzed by qRT-PCR. (B) and (D) Chicken DF-1 cells were treated with 10 μg/mL of poly (I:C) for 1, 3, 6, 12, and 24 h, and then the expressional level of *chLECT2* and *chIL1B* was analyzed by qRT-PCR. Each experiment was repeated for three times (n = 3). *chLECT2*, chicken leukocyte cell-derived chemotaxin 2; *chIL1B*, chicken interleukin 1B; poly (I:C), polyinosinic:polycytidylic acid; qRT-PCR, quantitative real-time polymerase chain reaction. Significant differences were determined by Tukey’s test, and bars with * symbol on top are the cases that significant differences were not found (p<0.05).

To understand the expression patterns of this gene, the transcriptional profile of *chLECT2* was investigated in chicken DF1 cells under the stimulation with TLR3 agonist, poly (I:C). To verify whether the expression of *chLECT2* gene is regulated by TLR3 signaling, the expression of *chIL1B*, which is known to be involved in TLR3 signaling, was also examined along with *chLECT2*. Stimulation with 10 μg/mL of poly (I:C) significantly upregulated both *chLECT2* and *chIL1B* expressions (Figure 2A, 2C). The levels of the *chLECT2* and *chIL1B* were increased significantly from 1 h after stimulation with 10 μg/mL of poly (I:C), and reached to maximum at 6 h after stimulation, which lasted to 24 h (Figure 2B, 2D).

### Effects of transcription inhibitors on *chLECT* mRNA expression

**Figure 3:**
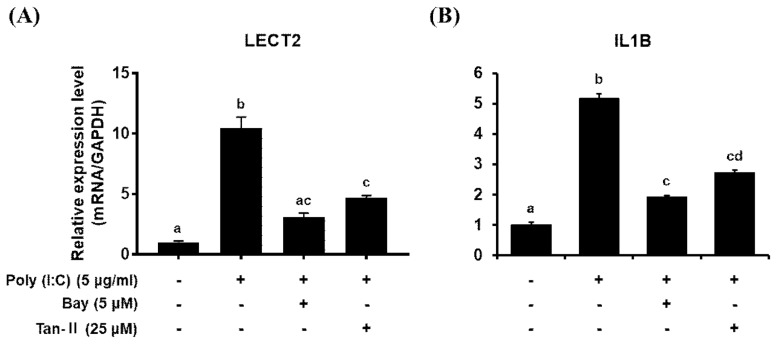
Effects of NFκB inhibitor (BAY11-7085; BAY) and AP-1 inhibitor (Tanshinone IIA; Tan-II) on the expressions of *chLECT2* (A) and *chIL1B* (B) after stimulation with poly (I:C) in chicken DF-1 cells. The cells were treated with or without 5 μg/mL of poly (I:C) for 6 h in the presence or absence of 5 μM of Bay or 25 μM of Tan-II. Values are mean±standard error (n = 3). NFκB, nuclear factor κB; AP-1, activated protein 1; *chLECT2*, chicken leukocyte cell-derived chemotaxin 2; *chIL1B*, chicken interleukin 1B; poly (I:C), polyinosinic:polycytidylic acid. Significant differences were determined by Tukey’s test, and bars with the same letter (^a–d^) on top are the cases that significant differences were not found (alpha<0.05).

To determine whether transcription factors NFκB and AP-1 are responsible for *chLECT2* transcription, DF-1 cells were pre-treated with NFκB inhibitor or AP-1 inhibitor before poly (I:C) stimulation (Figure 3). The expressions of both *chLECT2* and *chIL1B* were upregulated after treatment of 5 μg/mL of poly (I:C) without the inhibitors. However, in the presence of transcriptional inhibitors, i.e., BAY or Tan-II, the expressions of *chLECT2* and *chIL1B* were significantly suppressed compared to poly (I:C)-only treated control.

## DISCUSSION

In mammals, LECT2 is known as a hepatokine mediating obesity with insulin resistance and associated with the inflammatory response [23,24]. It was also reported that *LECT2* expression is increased in non-alcoholic fatty liver disease in association with metabolic syndromes such as abdominal obesity and lipid metabolism [12]. However, chLECT2 may play a different function compared to mammals. In previous report, we found the expression of *LECT2* increased from the kidney of broiler chickens fed with excessive calcium, above that required for the growth of chickens including body weight gain and eggshell formation [22]. The excessively high calcium doses in feed cause reduced body weight gain and lead to stress-induced diseases, even though it caused the increased expression of *chLECT2*. In this study, to investigate the role of *chLECT2* in chicken immune response and metabolic disturbance, the expression patterns of *chLECT2* under the stimulation of poly (I:C) revealed that the gene is involved in TLR3 signaling. Therefore, it is suggested that *chLECT2* may be related to the innate immune system upon the viral infection. Interestingly, Bornelov et al [25] recently reported that *chLECT2* expression in adipose tissue of layer hens is higher than broilers and suggested that the gene expression is related to the regulation of reproduction. Compared to this study, this means the function of LECT2 in chicken may vary tissue-specifically.

TLR3 plays a key role in initiating the immune response in chicken. As pathogens are detected by TLRs, the activation of transcription factors such as NF-κB and AP-1 is initiated. Afterwards, the transcriptional pathways stimulate the expression of pro-inflammatory cytokines including IL1B and type I interferon (IFN) and these downstream cytokines regulate the activity of immune-competent cells playing a role in innate immune defense systems. TLR3, as the receptor for viral dsRNA, triggers the antiviral immune pathway and induces the expression IFN which plays a critical role to antiviral activity.

Recently, Kamimura et al [26] reported that the transcription of the cytokine was induced by the transcriptional factors, NF-κB and AP-1 after stimulation of different TLR ligands in chicken vaginal cells. Also, they showed that the expression of *chIL1B* was suppressed by the inhibitors of the transcriptional factors, which are Bay for NF-κB, and Tan-II for AP-1. These results suggest that in chicken, poly (I:C) can stimulate the expression of *chLECT2* and *chIL1B* through the activation of both transcriptional factors, NF-κB and AP-1. Therefore, as for the typical TLR3 signal pathway, the expression of *chLECT2* can be induced by NF-κB and AP-1 under the stimulation of poly (I:C).

Besides mammals, several reports suggest that LECT2 plays important role in the inflammatory response and immune system in fish. The up-regulation of fish *LECT2* expression under bacterial infection has been reported in many fishes, including zebrafish with *Aeromonas salmonicida* [27], croceine croaker with *Vibrio alginolyticus* [28], asian seabass with *Vibrio harveyi* [29], lamprey with *Escherichia coli* [30]. In Asian seabass and croceine croaker, the expression of *LECT2* was significantly increased in liver and spleen with bacterial infection compared to healthy fish. As shown in Figure 1, the amino acid sequences of LECT2 orthologs have generally well conserved sequences but chLECT2 and duLECT2 have the same signal peptides and longer carboxy termini compared to other LECT2 orthologs. The phylogenetic analysis also revealed that chLECT2 is much closer to ducks and fishes than mammals. This might cause the similar but somewhat different function of chLECT2 compared to mammal orthologs.

Poly (I:C) is a prototypical class of pathogen-associated molecular patterns which are associated with pathogens. These molecules are recognized by TLRs and activate the innate immune system. Poly (I:C) is a stable synthetic dsRNA analogue which is generated during the viral replication, and is recognized by endosomally localized TLR3 ligand [31]. It is known that TLR3 prefers to recognize the synthetic poly (I:C) rather than virus-derived dsRNA, suggesting that TLR3 recognizes a unique dsRNA structure, different from other dsRNA-binding proteins [32]. The crucial role of TLR3 in poly (I:C) recognition was demonstrated by TLR3-knockout mice showing reduced inflammatory responses upon the treatment of poly (I:C) [33]. Many TLR3 effects rely on cells of the innate immune system that either express TLR3 or respond to inflammatory mediators that are produced upon TLR3 signaling.

In this study, we investigated the expression of *chLECT2* from DF-1 cells in response to TLR3 ligand and identified that NFκB and AP-1 transcription factors are indispensable for TLR3-mediated expression of chLECT2. The genetic information and the expression patterns of *chLECT2* revealed that *chLECT2* is evolutionarily much closer to ducks or fishes than the mammal orthologs. Because *LECT2* gene is known to be involved in inflammatory response, this report studied its biological role in chickens by investigating its expressional change under the treatment of TLR3 ligand and the inhibitors of transcriptional factors involved in TLR3 signaling. Also, the up-regulation of *chLECT2* expression after the stimulation of poly (I:C) suggests that this gene might be related to viral infection in chickens, and the significantly lower expressional level of *chLECT2* under the presence of transcriptional inhibitors, Bay and Tan-II verifies that the expression of this gene is involved in TLR3 signal pathway.

## CONCLUSION

Exploring the expression profiles of *chLECT2* and mechanisms by transcription factors of which activities are under TLR3 signals and regulate transcription of TLR3 target genes may provide fundamental knowledge of *LECT2* in chicken. Further study may confirm the contribution of chLECT2 in the innate immune system against viral diseases and the *chLECT2* gene is a promising candidate as a biomarker for infectious diseases in chickens.

## Figures and Tables

**Table 1:** Ensembl and amino acid sequence ID in the leukocyte cell-derived chemotaxin 2 gene of various species

Species	Scientific name	Ensembl ID	NCBI reference sequence ID
Chicken	*Gallus gallus*	ENSGALG00000006323	NM_205478.2
Duck	*Anas platyrhynchos*	ENSAPLP00000004471	KC250022.1
Human	*Homo sapiens*	ENSG00000145826	AB007546.1
Chimpanzee	*Pan troglodytes*	ENSPTRG00000017263	XM_517944.4
Mouse	*Mus musculus*	ENSMUSG00000021539	AB009687.1
Rat	*Rattus norvegicus*	ENSRNOG00000012189	NM_001108405.1
Cow	*Bos taurus*	ENSBTAG00000001247	AB001350.1
Pig	*Sus scrofa*	ENSSSCG00000014315	XM_021084783.1
Horse	*Equus caballus*	ENSECAG00000008324	XM_003362814.3
Zebrafish	*Danio rerio*	ENSDARG00000086483	DQ372604.1
Asian Seabass	*Lates calcarifer*	N/A	KF717364.1

**Table 2:** Quantitative polymerase chain reaction primers used for mRNA expression analysis

Target genes	Primers 5′-3′	Accession no
*IL1B*	F- GGA TTC TGA GCA CAC CAC AGT	XM_015297469.1
	R- TCT GGT TGA TGT CGA AGA TGT C	
*LECT2*	F- GAT ACG GCT GCG GCA ATT AC	NM_205478.2
	R- GCC CTT GTG CTT TTC TCC TTT	
*GAPDH*	F- TGC TGC CCA GAA CAT CAT CC	NM_204305.1
	R- ACG GCA GGT CAG GTC AAC AA	

*IL1B*, interleukin 1B; *LECT2*, Leukocyte cell-derived chemotaxin 2; *GAPDH*, glyceraldehyde 3-phosphate dehydrogenase.
